# Refeeding-Induced Brown Adipose Tissue Glycogen Hyper-Accumulation in Mice Is Mediated by Insulin and Catecholamines

**DOI:** 10.1371/journal.pone.0067807

**Published:** 2013-07-04

**Authors:** Christopher M. Carmean, Alexandria M. Bobe, Justin C. Yu, Paul A. Volden, Matthew J. Brady

**Affiliations:** 1 From the Committee on Molecular Metabolism and Nutrition, University of Chicago, Chicago, Illinois, United States of America; 2 Department of Medicine, Section of Endocrinology, Diabetes and Metabolism, University of Chicago, Chicago, Illinois, United States of America; University of Minnesota - Twin Cities, United States of America

## Abstract

Brown adipose tissue (BAT) generates heat during adaptive thermogenesis through a combination of oxidative metabolism and uncoupling protein 1-mediated electron transport chain uncoupling, using both free-fatty acids and glucose as substrate. Previous rat-based work in 1942 showed that prolonged partial fasting followed by refeeding led to a dramatic, transient increase in glycogen stores in multiple fat depots. In the present study, the protocol was replicated in male CD1 mice, resulting in a 2000-fold increase in interscapular BAT (IBAT) glycogen levels within 4–12 hours (hr) of refeeding, with IBAT glycogen stores reaching levels comparable to fed liver glycogen. Lesser effects occurred in white adipose tissues (WAT). Over the next 36 hr, glycogen levels dissipated and histological analysis revealed an over-accumulation of lipid droplets, suggesting a potential metabolic connection between glycogenolysis and lipid synthesis. 24 hr of total starvation followed by refeeding induced a robust and consistent glycogen over-accumulation similar in magnitude and time course to the prolonged partial fast. Experimentation demonstrated that hyperglycemia was not sufficient to drive glycogen accumulation in IBAT, but that elevated circulating insulin was sufficient. Additionally, pharmacological inhibition of catecholamine production reduced refeeding-induced IBAT glycogen storage, providing evidence of a contribution from the central nervous system. These findings highlight IBAT as a tissue that integrates both canonically-anabolic and catabolic stimulation for the promotion of glycogen storage during recovery from caloric deficit. The preservation of this robust response through many generations of animals not subjected to food deprivation suggests that the over-accumulation phenomenon plays a critical role in IBAT physiology.

## Introduction

The main adipose tissue types in mammals, WAT and BAT, are powerful, complementary regulators of systemic metabolism. WAT accommodates caloric excess by expanding to store triglycerides [1], [2] and compensates for caloric deficit through the mobilization of FFA. Conversely, in rodents, BAT undergoes adaptive thermogenesis to either burn excess calories following a meal [3] or for the maintenance of core body temperature [4]. During adaptive thermogenesis, energy from reducing agents, used to concentrate protons in the mitochondrial inter-membrane space, is expended in the form of heat due to the uncoupling protein 1-mediated dissipation of the proton concentration gradient. This process generates heat so effectively that rats with functional BAT can remain at near-freezing temperatures (6°C) for 10 days without succumbing to the cold [5]. During such a cold challenge, lipids are released from WAT into the circulation and then taken up and oxidized by BAT [5]–[7], completely re-organizing lipid distribution and gross adipose tissue physiology. In the context of this process, WAT and BAT comprise a highly-effective organ system for homeostasis. The recent reports of functional brown-like adipose tissue in human adults, as well as the potential to harness the thermogenic capacity of BAT to burn excess calories, make the modulation of BAT metabolism an attractive potential obesity therapeutic [8]–[10].

BAT activity is controlled by overlapping endocrine and neural inputs [4]. This regulation is made possible by extensive vascularization and sympathetic innervation of brown fat pads, enabling rapid access of both circulating metabolites and neural signals to the surface of brown adipocytes [11]–[13]. One major endocrine regulator of BAT metabolism, insulin, has been demonstrated as a critical component of both BAT-specific physiology and whole body glucose homeostasis as evinced by BAT-specific insulin receptor knockout studies [14]–[16]. Mice lacking the insulin receptor in BAT become progressively glucose intolerant and exhibit reduced IBAT mass [16]. Insulin action in BAT dramatically increases glucose uptake through canonical increases in GLUT4 expression and translocation to the plasma membrane [17], [18]. Much of the glucose taken up by BAT is used for anabolic processes such as glycogen storage, *de novo* free fatty acid synthesis, or triglyceride formation [19]. IBAT metabolism is also modulated by type II deiodinases that convert T_4_ to T_3_ [20], [21]. Ultimately, these endocrine hormones are complemented by signals from the sympathetic component of the central nervous system (CNS).

Central control of BAT metabolism plays a major role in the control of its metabolic activity [11], [20], [22], [23]. In the CNS, the preioptic chiasma and anterior hypothalamic nuclei integrate thermal status and insulin and leptin sensing to transmit a cholinergic signal along efferent paths [4]. Ultimately, the signal is propagated through sympathetic neural fibers that directly innervate BAT pads [24]. The catecholamines released from these fibers stimulate cell-surface β2- and β3-adrenergic receptors, activating an intensely-catabolic cellular program. The resulting cAMP-mediated signaling cascade increases BAT proliferation, GLUT1-mediated glucose uptake, lipolysis, and adaptive thermogenesis [4]. Sympathetic tone is powerful enough to drive significant IBAT glucose uptake even under markedly hypo-insulinemic conditions [22]. Upon adaptation to a cold environment, rats become hypo-insulinemic, yet their IBAT has one of the highest rates of glucose uptake of any tissue in the body [22], [25]. This elevated metabolic rate is manifested in part by a near-absence of the highly-labile glycogen that can normally be found in IBAT [26], [27]. Conversely, upon the transition from cold to warm, when sympathetic tone decreases drastically, IBAT fills with glycogen, associated with a rapid shift towards anabolic metabolism [26].

The control of IBAT glycogen metabolism is currently a controversial topic. Investigations conducted across the past 70 years have reported basal, fed-state IBAT glycogen levels in rats ranging from nearly undetectable [26], [28]–[31] through 0.5 mg/g tissue [32], and up to 2–5 mg/g tissue [27], [33]. Interestingly, notwithstanding differences in measured basal glycogen levels, investigators across many decades reported that a prolonged fast almost completely depletes IBAT glycogen and that *ad libitum* refeeding causes a robust over-accumulation [3], [28]–[30], [32]. This over-accumulation during refeeding with a mixed meal in rats can be hampered or intensified by decreasing or increasing, respectively, the carbohydrate content of the diet [29]. However, far less is known about the dietary control of glycogen dynamics in mouse IBAT. In the current study using wild-type outbred CD1 mice, IBAT glycogen over-accumulation occurred following refeeding after 24 hr of starvation, which was a shorter minimal starvation requirement than reported in historical studies. At peak levels, glycogen concentration in IBAT exceed those present in a fed liver, suggesting that glucose storage plays an important but underappreciated role in IBAT physiology.

## Experimental Procedures

### Mouse Treatment and Care

A wild-type CD1 mouse colony was maintained on a 12∶12-hr light:dark cycle (lights on at 06∶00) under specific pathogen-free conditions in the Knapp Center for Biological Sciences animal facility at the University of Chicago maintained at 22°C. Interactions with the mice performed during the dark phase were conducted under dim red lighting to minimize disruption of circadian cues. All animal procedures for this study were approved by the University of Chicago Institutional Animal Care and Use Committee (IACUC) and all efforts were made to minimize animal suffering. Throughout their lifetimes and during the appropriate phases of the described studies, mice were provided with irradiated Harlan Teklad Global 18% Protein Rodent Diet, referred to in this study as chow. Calories from protein, fat, and carbohydrates in the chow diet were 24%, 18%, and 58%, respectively (Harlan Laboratories, #2918).

### 5 Day Caloric Restriction/Refeeding

Male, wild-type, out-bred CD1 mice between 3 and 5 months of age were group-housed and acclimated to non-nutritive Aspen bedding with *ad libitum* access to food and water. After 5 days, mice were transferred to individual housing for 5 days on Aspen bedding, during which time average daily food intake was monitored. At the end of this individual housing acclimation, cages were changed (for total removal of nutrition), and a clump of dirty bedding from the acclimation cage was transferred with each mouse (to reduce scent stress). Mice were then allowed 60% of their ad libitum food intake administered daily at 19∶30, calculated by measuring total food intake during the final 3 days of the individual-housing acclimation time period. Removal of food and refeeding were conducted at 19∶30 to ensure that significant interactions with mice occurred while they were fully-awake. Mice were removed from the study if they became anorexic or severely dehydrated. After 5 days, mice were then sacrificed at 19∶30, or refed at 19∶30 and then sacrificed 4–48 hr later. Male mice were used because, although female mice responded similarly to 5 days of caloric restriction followed by refeeding during initial protocol establishment, the authors sought to avoid the possibility that any estrous cycle-related endocrine phenomena might confound the data.

### 72H- and 24H-Starvation/Refeeding

Male, wild-type, out-bred CD1 mice between 8 and 14 weeks of age were acclimated to non-nutritive Aspen bedding in groups of 2 or 3 with *ad libitum* access to food and water for 5 days. Food was then withdrawn at 19∶30, cages changed, and a clump of dirty bedding from the acclimation cage was transferred with each mouse. After 72 hr or 24 hr of starvation, mice were either sacrificed, or refed and then sacrificed at time points thereafter. The 24 hr starvation/refeeding protocol was also repeated with male WT C57BL/6J mice obtained from Jackson Laboratories and IBAT was harvested as described below for glycogen determination. All control mice (male, WT, CD1, 8–20 weeks of age) underwent the same acclimation procedures with unrestricted access to food, except that in some cases, fed-mouse cages were not changed after 5 days of acclimation on Aspen bedding prior to sacrifice.

### Starvation/Injection of Glucose

Male, wild-type, out-bred CD1 mice 8–9 weeks of age were acclimated and starved as described for the 24H-starvation/refeeding protocol. In lieu of refeeding, mice were given an intraperitoneal (IP) injection of 3 g glucose/kg of body weight (50% dextrose solution, Hospira Inc.) at 19∶30. Mice were then sacrificed 1 or 2 hr after injection.

### Starvation/Glucose-only Refeeding

Male, wild-type, out-bred CD1 mice 8–9 weeks of age were acclimated and starved as described for the 24H-starvation/refeeding protocol. In lieu of chow refeeding, mice were given a water bottle containing 10% glucose at 19∶30. Mice were then sacrificed 1 or 2 hr after provision of the glucose solution.

### Alpha-Methyl-Para-Tyrosine (AMPT) Injection Experiments

Male, wild-type, out-bred CD1 mice 8–9 weeks of age were acclimated and starved as described for the 24H-starvation/refeeding protocol. At 17∶30 (2 hr before scheduled refeeding), mice were injected with 300 mg/kg of AMPT (Sigma Corp. CAS: 672-87-7 dissolved in 0.9% saline). Mice were then allowed *ad libitum* access to chow at 19∶30. All mice were sacrificed 2, 4, or 6 hr following injection.

### Hyperinsulinemic, Hyperglycemic Clamp

The hyperinsulinemic, hyperglycemia clamp was performed as described previously [34], except that mice were fasted for 24 hr and then insulin was infused at 3 mU/kg/min and plasma glucose was maintained at 300 mg/dL.

### Animal Sacrifice

Mice were anaesthetized with Isoflurane vapor (Baxter Inc., NDC 10019-773-60) and cervical dislocation was performed. Tissues were immediately harvested in the following order as needed for each experiment: IBAT, whole blood from cardiac puncture, EPI, mesenteric adipose tissue, perirenal adipose tissue, and whole liver. Whole blood was immediately placed on ice in a 1.5-mL centrifuge tube for 15 to 30 minutes and then spun at 8,000 rpm for 10 minutes. Serum was then transferred to a fresh 1.5-mL centrifuge tube and stored at −80°C. All harvested tissues were immediately placed in liquid nitrogen and stored at −80°C.

### Glycogen Quantification (Adapted from [35])

#### Liver

Whole livers were homogenized by mortar and pestle while kept at below-freezing temperatures. An aliquot of each sample (15–40 mg tissue) was mixed with 1 mL of 10% trichloroacetic acid (Sigma, Cat. No. T0699) and immediately sonicated for 1.5 minutes (Sonics VibraCell Ultrasonic Processor - Amplitude setting: 50) and then placed on ice. Samples were spun at 12 k rpm for 15 minutes at 4°C to pellet all protein. The supernatant was split into 2 450-uL aliquots in 2-mL reserve-tip centrifuge tubes (Costar, Cat. No. 3213). These aliquots provided duplicate experimental measurements. 1.25 mL of 200-proof ethanol was added, and each sample was vortexed briefly and then placed at −80C. After at least 2 hr, each sample was spun at 12 kpm for 15 minutes at 4°C, the supernatant was decanted, and the inverted samples blotted on a paper towel briefly. 1.25 mL of 70% ethanol was added to wash each glycogen pellet, samples were inverted several times, and samples were spun again at 12 k rpm for 15 minutes at 4°C. Supernatant was decanted and samples were inverted and blotted on a paper towel and then dried in a Speedvac on medium heat for 30–45 minutes. Samples were resuspended in 125 uL of 0.2 M sodium acetate, pH = 4.6, and 8 ul of amyloglucosidase (Sigma, CAS 9032-08-0, 30 mg/mL in 0.2 M sodium acetate, pH = 4.6). Samples were incubated at 42°C with 100 rpm shaking in the dark overnight and then measured by glucose oxidase assay as described previously [36].

### Adipose Tissues

Frozen adipose tissues (IBAT: 15–60 mg; perirenal, mesenteric, and subcutaneous adipose tissues: 80–150 mg; EPI: 80–250 mg) were individually mixed with 500 uL of 10% trichloroacetic acid (Sigma, Cat. No. T0699) and immediately sonicated for 1.5 minutes at medium power (Sonics VibraCell Ultrasonic Processor - Amplitude setting: 50) and then placed on ice. 350 uL of chloroform was added to extract lipids, and each sample was vortexed for 15 seconds. Samples were spun at 12 k rpm for 15 minutes at 4C. 450 uL of supernatant was transferred to 2-mL reserve-tip centrifuge tubes, 1.25 mL 200-proof ethanol was added, and each sample was vortexed briefly then placed at −80C for at least 2 hr. Samples were processed from this point identically to liver samples as described above.

### Radioactive Samples

Samples collected from the hyperinsulinemic, hyperglycemic clamp were radioactive and therefore were not sonicated. Adipose tissues were treated exactly as described above, except that they were homogenized by glass dounce homogenizer instead of sonication. Control, non-radioactive samples processed by each homogenization method confirmed that the methods produced similar results (data not shown).

### Tissue Histology

Formalin-fixed brown adipose and liver tissues were mounted in paraffin, sectioned, and stained using the Periodic Acid-Schiff method to visualize carbohydrate, with H&E counterstain, by the University of Chicago Human Tissue Resources Center.

### Serum Measurements

Serum insulin was determined by enzyme-linked immunosorbent assay (ALPCO Mouse Ultrasensitive Insulin ELISA kit, Cat. No 80-INSMSU-E01). Serum glucose was determined by the colorimetric glucose oxidase activity assay as described previously [35].

### Statistical Analysis

Each comparison made between two treatments or conditions was performed with a 2-tailed Student’s t-test. Significance was set at *p<0.05*.

## Results

### Circadian Oscillation in Liver, but not Adipose Tissue, Glycogen Levels

**Figure 1:**
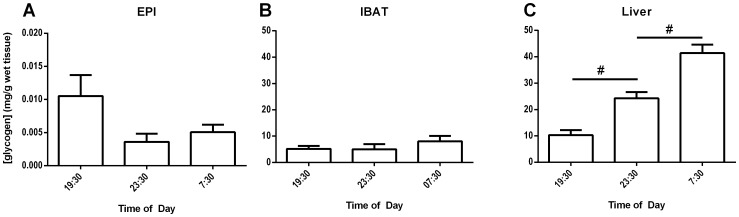
Fed-mouse tissue glycogen concentrations. Group-housed, fed, male, wild-type, CD1 mice were sacrificed at 19∶30, 23∶30, or 07∶30 and tissues were harvested and snap-frozen in liquid nitrogen. Tissue glycogen concentrations were measured in epididymal adipose tissue (EPI) (**A**), interscapular brown adipose tissue (IBAT) (**B**), and liver (**C**). Error bars are ±SEM. Statistical comparisons for each tissue were made between time points using a 2-tailed Student’s t-test. ^#^, p<0.001. 10–30 mice were used for each time point.

Measurements of basal, fed-state IBAT, epididymal WAT (EPI), and liver glycogen levels were taken across the 12-hr awake-period to address the possibility that mouse adipose tissues underwent diurnal fluctuations. Neither EPI nor IBAT glycogen levels changed significantly between 19∶30 and 07∶30 in male CD1 mice (Fig. 1A, B, respectively). Therefore, all values for each adipose tissue measured across time points were averaged. Mean EPI and IBAT glycogen were 0.006 mg/g tissue and 5.86 mg/g tissue, respectively. Basal IBAT glycogen was slightly more concentrated than published reports of muscle tissue glycogen [37], making it the second most glycogen-rich depot after liver in the body. Mean liver glycogen accumulated significantly during the feeding period across the dark cycle from 10.27 mg/g wet tissue to 41.40 mg/g wet tissue (Fig. 1C), in agreement with prior literature [27], [38]. Additionally, the mean concentration of fed-state IBAT glycogen was consistently within 1 order of magnitude of fed liver glycogen (Fig. 1B vs. Fig. 1C).

### Prolonged Partial Caloric Restriction Followed by Refeeding Induces Glycogen Over-accumulation

**Figure 2:**
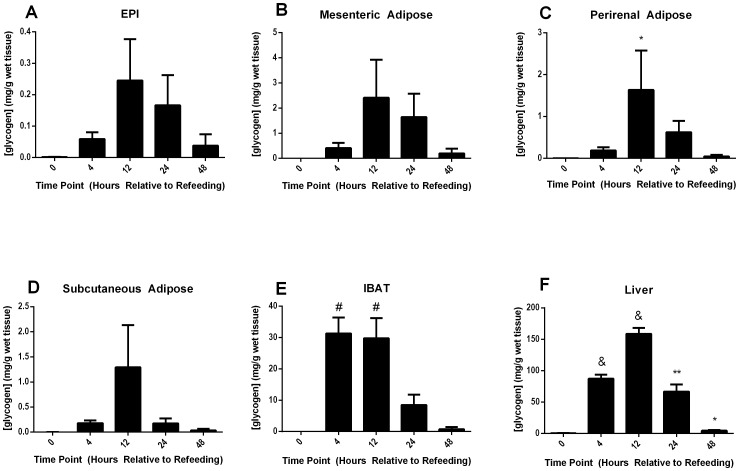
Tissue glycogen during 5 days of partial fasting and refeeding. Male, wild-type, CD1 mice were fed 60% of their normal daily intake of chow once daily for 5 days and then sacrificed (0) or allowed to refeed *ad libitum* 4–48 hr before sacrifice (4–48). Upon sacrifice, tissues were harvested and snap-frozen in liquid nitrogen. Tissue glycogen was measured in EPI (**A**), mesenteric adipose tissue (**B**), perirenal adipose tissue (**C**), subcutaneous adipose tissue (**D**), IBAT (**E**), and the liver (**F**). Error bars are ±SEM. Statistical comparisons were made between the partial-fasting time point (0) and each refeeding time point (4–48) for each tissue using a 2-tailed Student’s t-test. *, p<0.05; **, *p<0.01*; ^#^, *p<0.001*; ^&^, *p<0.0001.* Fasted time points utilized 2 mice. All other time points were obtained from 4–8 mice.

Historical rat-based studies utilized prolonged caloric restriction (1–2 weeks) followed by *ad libitum* refeeding to activate IBAT- and EPI-glycogen over-accumulation [28], [29]. A protocol was adapted from these previous studies to define changes in mouse adipose tissue glycogen levels under similar conditions. Mice were calorically restricted, receiving 60% of their individual *ad libitum* food intake for 5 days, and then sacrificed or allowed to refeed *ad libitum* 4–48 hr prior to sacrifice. Tissue glycogen concentrations were measured along this time course for WAT depots (EPI, mesenteric, perirenal, and subcutaneous), IBAT, and liver (Fig. 2A–F, respectively). All harvested tissues except IBAT revealed a peak glycogen accumulation of 12 hr post-refeeding, followed by a decline in glycogen through the final 48 hr time point measured. EPI, which had been the most widely studied depot for glycogen hyper-accumulation in mice and rats had the lowest absolute levels of glycogen, yet displayed a 40-fold increase in glycogen stores upon refeeding relative to the calorically-restricted state (Fig. 2A). Peak EPI glycogen trended towards significance when compared to the partially-fasted 0 hr glycogen measurements (p = 0.08). Mesenteric, perirenal, and subcutaneous adipose tissues all showed similar magnitudes of glycogen accumulation, with peak values between 1–3 mg glycogen/g tissue (Fig. 2 B, C, D, respectively). As with the EPI depot, the 12 hr glycogen measurement in mesenteric and subcutaneous adipose tissues trended towards significance when compared to their respective partially-fasted 0 hr glycogen measrurements (p = 0.10 and p = .05 respectively). Perirenal adipose tissue 12 hr after the onset of refeeding was significantly elevated relative to the partially-fasted 0 hr time point. Interestingly, glycogen storage was slightly accelerated in IBAT relative to other tissues, with a peak occurring at 4 to 12 hr of refeeding. Glycogen levels in this time period were elevated more than 2000-fold compared to the end of the partial fast (Fig. 2E), and reached concentrations comparable to the fed liver (Fig. 1C). Hepatic glycogen storage at its peak exceeded all other measured values, reaching 158.7 mg glycogen/g tissue (Fig. 2F).

**Figure 3:**
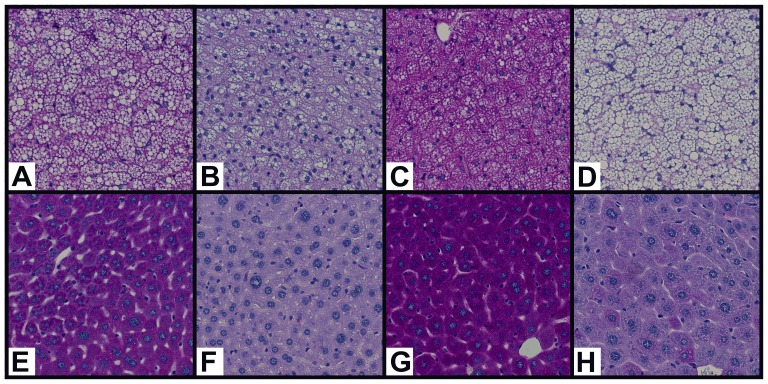
Histological visualization of glycogen storage in IBAT (top row) and liver (bottom row) using Periodic Acid Schiff staining. Male, wild-type, CD1 mice were either fed ad libitum (**A**, **E**), fed 60% of their normal daily intake of chow once daily for 5 days and then sacrificed (**B**, **F**), or fed 60% of their normal daily intake of chow once daily for 5 days and then allowed to refeed *ad libitum* 4 hr (**C**, **G**) or 48 hr (**D**, **H**) before sacrifice. IBAT (**A–D**) and liver (**E–H**) were harvested and immediately placed in formalin fixative solution. Fixed samples were mounted in paraffin, sectioned, and stained using Periodic Acid Schiff staining with Hematoxylin and Eosin counter-staining by the University of Chicago Human Tissue Resources Center.

Periodic Acid Schiff staining of glycogen was used to visually confirm quantitative results in IBAT and liver (Fig. 3). Glycogen content was visually lower in IBAT after 5 days of caloric restriction (Fig. 3B) compared to fed controls (Fig. 3A), followed by a massive over-accumulation only 4 hr after refeeding (Fig. 3C). It was also noted that IBAT lipid droplets appeared relatively depleted after 5 days of partial fasting (Fig. 3B) compared to the fed state (Fig. 3A), and that 4 hr into refeeding, concomitant with the over-accumulation of glycogen, lipid droplets appear to increase in size (Fig. 3C). Finally, 48 hr after the onset of refeeding (Fig. 3D), the tissue appeared more replete with lipid droplets than in the fed state control (Fig. 3A). PAS staining of the liver yielded a similar pattern of glycogen storage (Fig. 3E–H).

### Total Starvation Followed by Refeeding Induces Glycogen Over-accumulation

**Figure 4:**
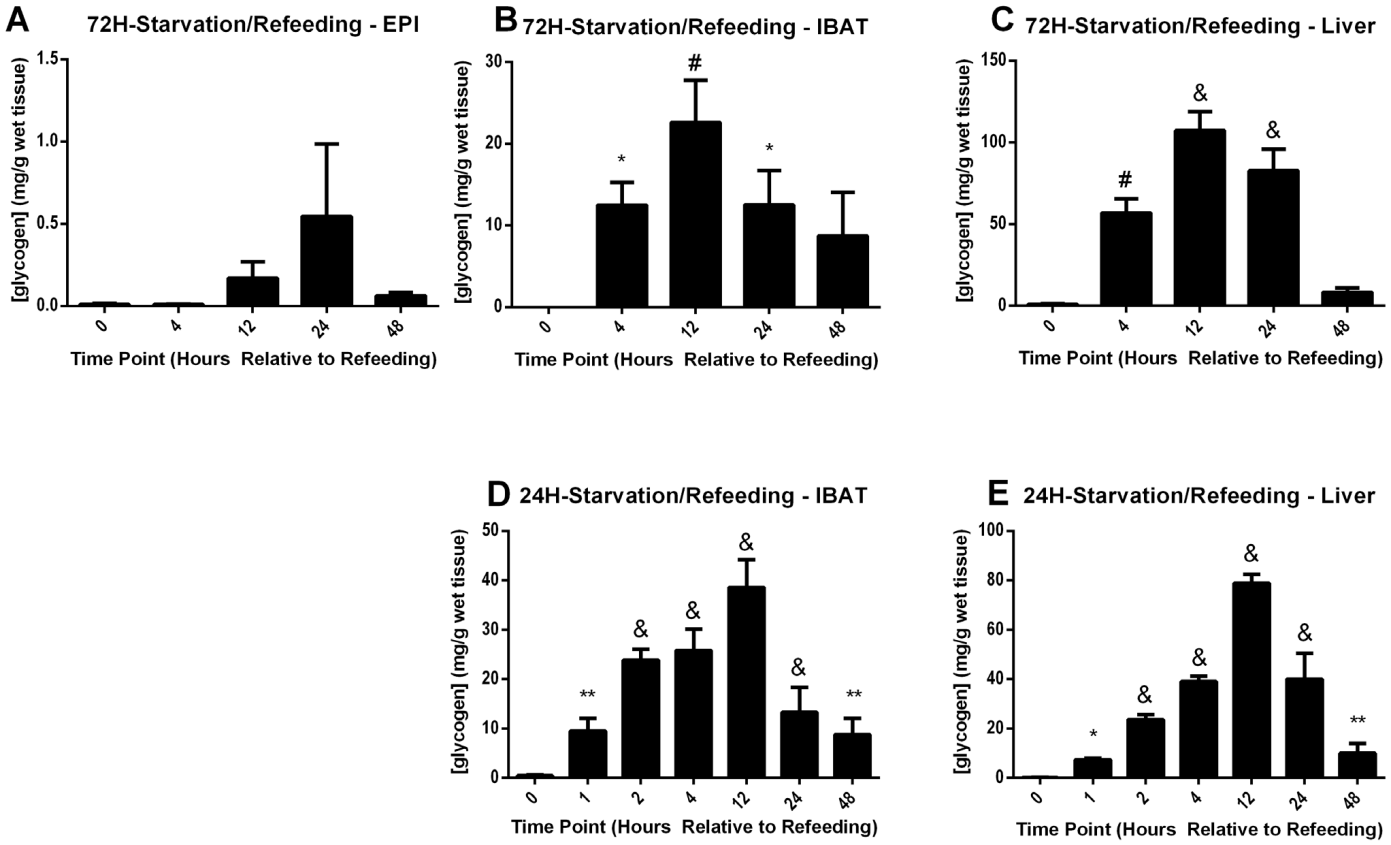
Tissue glycogen during refeeding after total starvation. Male, wild-type, CD1 mice were starved for either 72 hr (**A–C**) or 24 hr (**D,E**) and then sacrificed (0) or allowed to refeed *ad libitum* 1–48 hr before sacrifice (1–48). Tissues were harvested and snap-frozen in liquid nitrogen. Tissue glycogen was measured in EPI (**A**), IBAT (**B, D**), and liver (**C, E**). Error bars are ±SEM. Statistical comparisons were made between the starved time point (0) and each refeeding time point (1–48) for each tissue using a 2-tailed Student’s t-test. *, p<0.05; **, *p<0.01*; ^#^, *p<0.001*; ^&^, *p<0.0001.* Fasting time points utilized 3–5 mice, except 24 hr fasting BAT glycogen which utilized 19 mice. All refed measurements were taken from 3–9 mice except 24 hr fasted/refed 2 hr BAT time point, which was measured in 15 mice.

Because mesenteric, perirenal, and subcutaneous adipose tissues each consist of a variable combination of white and ‘beige’ adipocytes [5], [39], glycogen storage might have been representative of an admixture of those adipocytes. To avoid this potential confounder of data interpretation, further experiments were limited to the mostly-white adipocyte EPI depot and the mostly-brown adipocyte IBAT depot, and compared to results in the liver. Due to the complicated and protracted nature of the partial caloric restriction protocol, a variety of alternative protocols were tested for their ability to replicate the glycogen spikes in WAT and IBAT. Following 72 hr of complete starvation, mice were sacrificed or given *ad libitum* access to food and sacrificed 4–48 hr later. Refeeding activated a glycogen over-accumulation profile in EPI, IBAT, and liver (Fig. 4 A–C, respectively) that was comparable to the time courses and peak values achieved during the 5 day partial fasting protocol, although the nearly-significant (p = 0.09 versus 0 hr) peak in EPI was delayed until 24 hr after refeeding (Fig. 4A). Due to the low absolute range of EPI glycogen deposition, the remainder of this study focused on IBAT and liver.

Next, more acute 24 hr and 16 hr starvation protocols were tested, and a more detailed time course following refeeding was performed to examine IBAT and liver glycogen storage. 24 hr of starvation followed by refeeding induced robust and significant glycogen over-accumulation in both tissues examined (Fig. 4D, E, respectively), however the 16 hr protocol displayed reduced consistency of over-accumulation and altered kinetics (data not shown). The 24H-starvation/refeeding protocol was also repeated using the in-bred C57BL/6J strain of mice, and yielded IBAT glycogen over-accumulation during refeeding (data not shown), demonstrating that the response was not strain-specific. For these reasons, the 24H-starvation/refeeding protocol was selected for further experiments as a representative model of starvation followed by refeeding. IBAT glycogen concentrations prior to refeeding during the 72H-starvation/refeeding (Fig. 4B), 24H-starvation/refeeding (Fig. 4D), and 5d-partial-fast/refeeding (Fig. 2E) protocols were not significantly different. Peak IBAT glycogen concentrations after refeeding across the 5d-partial-fast/refeeding (Fig. 2E), 72H-starvation/refeeding (Fig. 4B), and 24H-starvation/refeeding (Fig. 4D) were also not significantly different from each other.

### Hyperglycemia Alone is Insufficient to Induce IBAT Glycogen Accumulation Following Starvation

Once 24 hr of starvation followed by *ad libitum* refeeding was identified as a suitable protocol for studying glycogen hyper-accumulation, the roles of potential nutritional, endocrine, and neurological cues were assessed. The ability of hyperglycemia to activate IBAT and liver glycogen deposition in 24 hr-starved mice was first tested. A 3 g/kg IP bolus of glucose solution failed to initiate IBAT glycogen deposition in mice starved for 24 hr (Table 1, 24H-SIG). The injection resulted in the rapid accumulation of liver glycogen matching the time course and magnitude of liver glycogen in 24 hr starved/refed mice, as well as elevated serum glucose levels above those observed during refeeding (Table 1). In agreement with other reports [40], [41], glucose injection did not stimulate any significant increase in circulating insulin at the intervals measured (Table 1, 24H-SIG), making this a clear determination of the insufficiency of hyperglycemia alone to induce glycogen over-accumulation in IBAT. Fasted control mice that were allowed to refeed after receiving an IP injection of saline solution did not display any significantly-lower IBAT glycogen, liver glycogen, or serum glucose or insulin compared to un-injected, refed mice (Table 1, 24H-SSR), confirming that the IP injection itself does not suppress the natural IBAT and liver glycogen hyper-accumulation during refeeding. The act of injection, therefore, was not likely responsible for the failure of glucose to induce IBAT glycogen deposition.

**Table 1:** Comparison of tissue glycogen and serum parameters between fed, 24 hr-starved, and chow-refed or alternative interventions.

		IBAT Glycogen (mg/g tissue)				Liver Glycogen (mg/g tissue)				Serum Glucose (mg/dL)				Serum Insulin (ng/mL)			
Condition	TP H	Mean	SEM	p value vs Fed	p value vs 24H-S	Mean	SEM	p value vs Fed	p value vs 24H-S	Mean	SEM	p value vs Fed	p value vs 24H-S	Mean	SEM	p value vs Fed	p value vs 24H-S
**Fed**	0	5.18	1.08	–	0.001	10.27	1.94	–	<0.05	202.44	18.99	–	<0.0001	2.42	1.28	**–**	NS
**24H-S**	0	0.41	0.18	0.001	–	0.1	0.07	<0.05	–	73.03	9.89	<0.0001	–	0.24	0.15	NS	**–**
**24H-SR**	1	9.43	2.66	NS	<0.0001	7.36	0.63	NS	<0.0001	151.5	5.56	<0.05	<0.0001	6.99	1.06	<0.01	<0.01
	2	23.86	2.19	<0.001	<0.0001	23.59	2.09	<0.01	<0.0001	144.6	8.13	<0.05	<0.0001	9.43	1.43	<0.01	<0.01
**24H-SSR**	1	18.4	3.17	<0.001	<0.0001	10.64	0.73	NS	<0.0001	159.37	15.64	NS	<0.001	7.67	1.02	<0.01	<0.001
	2	24.99	2.61	<0.001	<0.0001	22.42	1.65	<0.05	<0.0001	142.56	10.39	NS	<0.01	15.4	3.31	<0.01	<0.01
**24H-SIG**	1	0.26	0.23	<0.05	NS	8.33	0.75	NS	<0.0001	406	30.3	<0.0001	<0.0001	0.09	0.02	<0.05	NS
	2	0.03	0.01	NS (0.06)	NS	20.22	2.53	<0.05	<0.0001	216	17.3	NS	<0.0001	0.08	0.02	NS	NS
**24H-SOG**	1	4.29	1.1	NS	<0.0001	10.8	1.47	NS	<0.0001	337.14	39.53	<0.01	<0.0001	2.21	0.53	NS	<0.05
	2	3.87	0.82	NS	<0.0001	29.71	1.87	<0.0001	<0.0001	238.34	22.87	NS	<0.0001	2.33	0.47	NS	<0.05

Abbreviations used for experiments (top to bottom rows): Control mice with *ad libitum* access to food (Fed); 24 hr starvation (24H-S); 24 hr starvation followed by chow refeeding (24H-SSR); 24 hr starvation followed by saline injection and *ad libitum* chow refeeding (24H-SSR); 24 hr starvation followed by an IP injection of 3 k/kg glucose (50% dextrose solution) (24H-SIG); 24H-starvation followed by *ad libitum* access to 10% oral glucose solution (24H-SIG). T.P. = Time point of sacrifice relative to the cessation of total starvation (either introduction of food (24H-SR), IP injection of glucose (24H-SIG), or provision of oral glucose solution (24H-SOG)). IBAT glycogen, liver glycogen, serum glucose, and serum insulin were measured. Statistical significance was evaluated by comparing individual measurements to the respective *ad libitum* fed state and separately to the 24 hr-starved state. One clear outlier for 24 hr-starved liver glycogen was removed from consideration (liver glycogen = 24.88 mg/g tissue). SIG, SOG treatment data were obtained from 5–13 mice for each time point, and SSR data were obtained for 3–6 mice.

### Ad Libitum Glucose Feeding after Starvation Induces Glycogen Deposition, but not Over-accumulation

The inability of injected glucose to raise serum insulin levels despite inducing marked hyperglycemia raised the possibility of a potential incretin effect in the refeeding response [42], [43]. To determine if the route of delivery of glucose to the animal was important for IBAT glycogen accumulation, mice were starved for 24 hr, provided water bottles containing 10% glucose solution, and then sacrificed either 1 or 2 hr later. IBAT glycogen accumulated to fed-state levels after 1 hr of glucose feeding and persisted through 2 hr, but glycogen over-accumulation did not occur (Table 1, 24H-SOG). Glucose feeding produced transient hyperglycemia and insulin levels of 2.2–2.3 ng/mL (not significantly different from the fed state). These data added evidence supporting the hypotheses that hyperglycemia *per se* was not sufficient to stimulate IBAT glycogen over-accumulation as observed with *ad libitum* refeeding, and that insulin was likely a primary driver of glycogen deposition.

### Normo-insulinemia with Hyperglycemia Induces glycogen Accumulation, but not Over-accumulation Following Starvation

**Figure 5:**
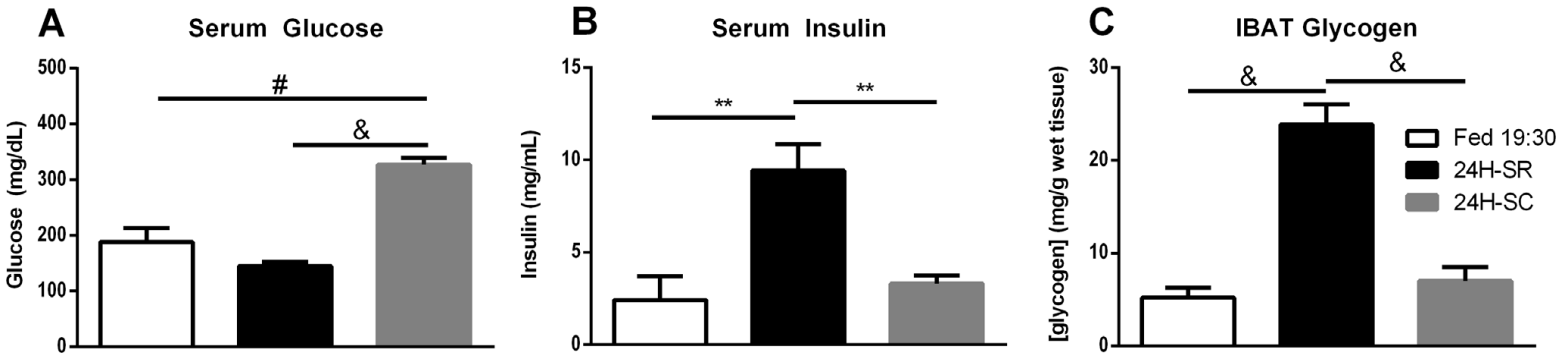
IBAT glycogen and serum parameters after 24 hr starvation and a hyperinsulinemic, hyperglycemic clamp. Male, wild-type, CD1 mice were starved for 24 hr and then subjected to a 2 hr hyperinsulinemic, hyperglycemic clamp (24H-SC) in which glucose was clamped at 300 mg/dL and insulin was infused at a rate of 3 mU/kg/min. At the end of the clamp, mice were sacrificed, serum was collected, and tissues were collected and snap-frozen in liquid nitrogen. Final serum glucose (**A**), serum insulin (**B**), and IBAT glycogen (**C**) were compared to those of fed mice sacrificed at 19∶30 (Fed) and 24 hr starved/refed mice sacrificed 2 hr after the onset of refeeding (24H-SR). Error bars are ±SEM. Statistical comparisons were made between experimental conditions using a 2-tailed Student’s t-test. **, *p<0.01*; ^#^, *p<0.001*; ^&^, *p<0.0001*. All measurements represent 8–9 mice, except Fed 19∶30 and 24H-SR IBAT glycogen, obtained from 30 and 15 mice, respectively.

Though altering the route of glucose administration failed to induce glycogen over-accumulation, it did succeed in restoring IBAT glycogen and serum insulin levels to those of the fed state (Table 1, 24H-SOG). It was unknown whether fed-state insulin, given adequate circulating glucose, could induce the repletion of IBAT glycogen in the absence of other physiological factors associated with refeeding. To assess the possibility that insulin was the primary driver of glycogen repletion, mice starved for 24 hr received a constant intravenous infusion of insulin and glucose for 2 hr using a hyperinsulinemic, hyperglycemic clamp and were then immediately sacrificed. Insulin was infused at a rate of 3 mU/kg/min for the duration, plasma glucose levels were brought above 300 mg/dL (Fig. 5A), and circulating levels of insulin at the end of the clamp were 3.3 ng/mL (Fig. 5B). IBAT glycogen levels at the end of the clamp were 7.01 mg/g wet tissue (Fig. 5C). Final IBAT glycogen and circulating insulin were each significantly lower than that of 24H-starved/refed mice sacrificed after 2 hr of refeeding, but not significantly different from fed mice sacrificed at 19∶30. These infusions effectively recapitulated fed-state serum insulin in fasted mice, and induced marked hyperglycemia without the ingestion of calories, demonstrating that insulin and hyperglycemia, in the absence of meal-associated gut hormones or any cephalic reaction to the act of refeeding, were sufficient to drive IBAT glycogen storage to fed-state levels in starved mice.

Further tests were conducted using an *in vitro* culture system, in which minced IBAT from 24 hr fasted mice was placed in Dulbecco’s Modified Eagle Medium with or without serum and exposed to a broad range of insulin, glucose, and/or T_3_ doses. Exposure for 1–4 hr of any combination of these agents failed to induce any glycogen accumulation (data not shown). These *in vitro* data indicated that there was likely some other physiological modifier of IBAT glycogen metabolism other than direct hormone or glucose signaling.

### Catecholamine Production is Necessary for Refeeding-induced IBAT Glycogen Storage

**Figure 6:**
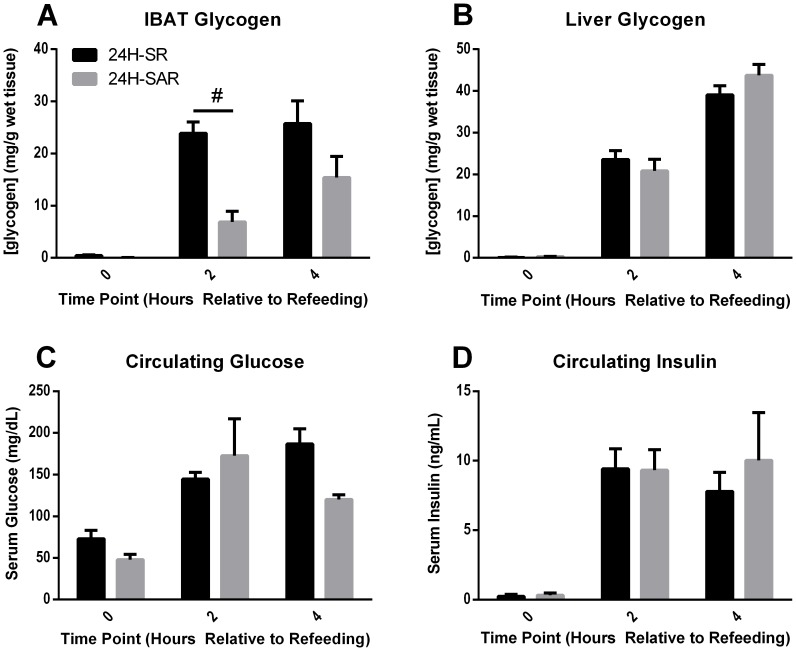
Effects of catecholamine-synthesis inhibition on tissue glycogen and serum parameters during starvation and refeeding. Male, wild-type CD1 mice were used for these experiments. 24H-SR mice were starved for 24 hr and either sacrificed (0) or allowed to refeed *ad libitum* 2–4 hours and then sacrificed (2, 4). 24H-SAR mice were starved for 24 hr, but also received an IP injection of 300 mg/kg AMPT in 0.9% NaCl 2 hr prior to refeeding. 24H-SAR mice were either sacrificed following the full 24 hr starvation (0), or allowed to refeed 2 or 4 hr prior to sacrifice (2, 4 respectively). Upon sacrifice, tissues were snap-frozen in liquid nitrogen and serum was collected. Glycogen was measured in IBAT (**A**) and liver (**B**), and circulating glucose (**C**) and insulin (**D**) were also measured. Error bars are ±SEM. Statistical comparisons were made between experimental conditions using a 2-tailed Student’s t-test. ^#^, *p<0.001*. Measurements for each time point from AMPT-injected mice were obtained for 7–12 mice.

**Figure 7:**
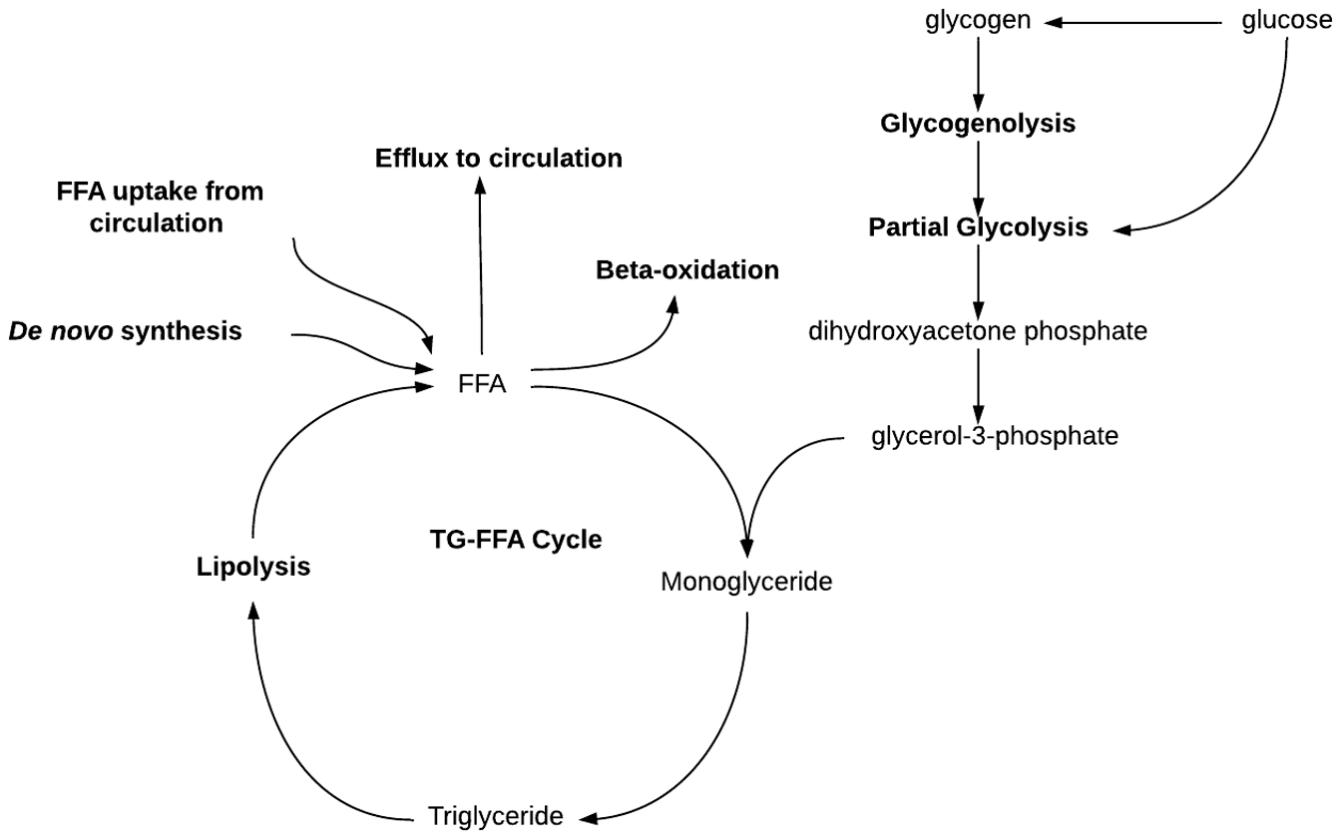
Mechanism by which glycogen stores may enhance free fatty acid esterification in adipose tissues during refeeding. During the first several hours of refeeding, hyperaccumulation of intracellular BAT glycogen occurs. The eventual glycogenolytic and subsequent glycolytic processing of glycogen stores may generate elevated levels of glycerol-3-phosphate, a precursor to monoglyceride formation. An increase in this precursor may enhance the rate of esterification of free fatty acids (FFA), both from *de novo* synthesized sources as well as FFA recently liberated from stored neutral lipids. This process may greatly enhance lipid repletion during refeeding in order to maximize recovery from the starvation state.

IBAT is thoroughly innervated by the sympathetic nervous system and centrally controlled by the ventromedial hypothalamus [44]. This signaling pathway is responsible for integrating both nutritional and thermoregulatory signals, resulting in the potential for marked changes in IBAT metabolic activity under physiological conditions [4]. To investigate the role of the sympathetic nervous system in the control of IBAT and liver glycogen, starved mice were injected with 300 mg/kg AMPT, a catecholamine synthesis inhibitor, 2 hr prior to refeeding. It had repeatedly been demonstrated that AMPT administered intraperitoneally at this dosage dramatically reduced sympathetic tone through the inhibition of catecholamine production, resulting in lower epinephrine and norepinephrine levels within whole IBAT fat pads [20], [45], [46]. 2 hr after the onset of refeeding, IBAT in AMPT-injected mice accumulated glycogen to a concentration of only 6.88 mg/g wet tissue, 70% less than un-injected, refed mice (Fig. 6A). Mean per-mouse food intake for refed mice and injected/refed mice were nearly identical at 1.70 g and 1.67 g, respectively. After 4 hr, AMPT-injected mice had trended towards significantly less IBAT glycogen (p = 0.10), accumulating 40% less than un-injected, refed mice (Fig. 7A).

Throughout the experiment, AMPT injection did not induce significant differences in liver glycogen, circulating glucose, or circulating insulin (Fig. 6B–D, respectively). These AMPT-injection data suggested that intact catecholamine signaling played a significant role during the initial hyper-accumulation of IBAT glycogen, eventually becoming less physiologically-relevant by 4 hr following refeeding.

## Discussion

The ability of mammals to enhance energy reserve repletion during refeeding after starvation in a variety of mammalian species has been independently verified by several investigators [47]–[50] and arguably involves every major organ system in the body. Centrally-mediated orexigenic signals induce marked hyperphagia, ensuring adequate caloric intake upon food availability. Upon refeeding, carbohydrate and lipids stimulate incretin secretions from the gut, which act on islets of Langerhans to elevate circulating insulin and suppress glucagon production, promoting anabolic processes in the periphery [42], [43]. Simultaneously, the CNS coordinates a tissue-specific sympathetic response to reduce torpor and hepatic gluconeogenesis [51], [52]. Animals in this phase of recovery from caloric deficit exhibit a shift from oxidative to glycolytic metabolism, with increased peripheral glucose uptake, *de novo* lipogenesis, and storage of dietary carbohydrate and lipids [53]–[56]. Given these complex, inter-related processes, it has been historically difficult to tease out the specific regulators of the glycogen over-accumulation phenomenon. This study examined for the first time the minimal caloric restriction requirements for eliciting refeeding-induced glycogen over-accumulation in murine adipose tissue. A 2000-fold over-accumulation of IBAT glycogen occurred without transgenic modification or pre-conditioning, and peaked similarly across caloric restriction protocols ranging in length from 24 to 120 hr.

Previous studies had described significant IBAT-metabolic responsiveness to glucose infusion, stemming, at least in part, from CNS-mediated integration of direct glucose-sensing in the VMH as well as vagal afferent signals from hepato-portal glucose sensors [57]–[59]. Given that the VMH-mediated sympathetic tone is one of the major regulators of IBAT metabolic activity [4], it was surprising that such elevated glucose levels by IP injection failed to induce IBAT glycogen over-accumulation following starvation. Thus, neither glucose sensing at the cell surface, nor indirect sensing through the CNS or afferent glucose sensors, were capable *per se* of activating IBAT glycogenesis during refeeding in mice. It is still unclear whether a rise in serum glucose above starvation-state levels is necessary for IBAT glycogen hyper-accumulation, but an increase in circulating glucose can be ruled out as a primary, independent driver of glycogen storage.

Interestingly, glucose injection activated an increase in hepatic glycogen content similar to that observed for 24H-starved/refed mice, despite a near-complete absence of circulating insulin. This observation is consistent with previous reports which determined that hyperglycemia promoted hepatic glycogenesis even when insulin levels were suppressed using anti-insulin serum or were highly variable [60], [61]. The results presented here indicate that hyperglycemia alone is sufficient to induce the observed hepatic glycogenesis in 24H-fasted mice, and that an elevation of hepatic glycogenesis in the starved-state is not dependent upon a persistent rise in circulating insulin.

Changing the route of glucose administration from injection to oral ingestion of glucose solution stimulated the repletion of IBAT glycogen to fed-state levels. Oral administration drove elevated insulin levels on-par with those of fed mice. This result suggested that insulin may have been the primary factor during refeeding responsible for induction of glycogen repletion, however there were other possibilities to consider. Refeeding induces the release of glucose-dependent insulinotropic peptide, and glucagon-like-peptide 1, from nutrient-sensing cells lining the gut [42], [43]. In addition to fulfilling their recognized roles as stimulators of insulin secretion, it was possible these hormones were directly facilitating glycogen repletion. However, the hyperinsulinemic, hyperglycemic clamp elevated circulating insulin and glucose independently of altered gut hormones levels. The clamp stimulated IBAT glycogen accumulation to similar levels as those seen during oral glucose feeding, demonstrating that insulin was likely the primary driver of glycogen repletion during glucose refeeding. This clarified that glucose ingestion likely served two purposes: providing substrate for glycogen repletion and stimulating insulin secretion. This is not entirely unexpected, as IBAT has long been recognized as an insulin-sensitive tissue [4], [31].

In addition to insulin, IBAT metabolic activity is tightly controlled by adrenergic hormones, primarily through the direct sympathetic-neuronal release of norepinephrine and epinephrine onto brown adipocytes [4], [11], [20]. Glycogen storage in IBAT has been shown to change inversely with proportion to sympathetic tone, which has been achieved in previous studies by manipulation of housing temperature or surgical intervention [11], [20], [22], [24], [26], [44]. In the present study, blocking catecholamine production and thereby reducing sympathetic tone reduced refed-state IBAT glycogen by 70% 2 hr following refeeding (Fig. 6A). This at first appeared to contradict the published reports that a reduction in sympathetic tone was associated with elevated IBAT glycogen levels. This disparity can be explained by the dynamic balance between anabolic and catabolic signals in IBAT. The sympathetic CNS increases GLUT1-mediated glucose uptake and up-regulates catabolic processes, while insulin promotes GLUT4-mediated glucose uptake and stimulates anabolic processes. It is possible that the glycogenic response to refeeding during the first few hr of refeeding required a baseline of catecholamine signaling to promote rapid glucose uptake, combined with insulin stimulation to induce an intracellular program favoring glycogen storage. When viewed as a coordinated response to metabolic demand, the interplay between insulin and sympathetic tone with respect to IBAT glycogen dynamics appears consistent across both caloric and thermal challenges. CNS activity and insulin appear to be acting synergistically to promote elevated glucose uptake and glycogenesis, thereby enhancing the rate of utilization of recently-ingested calories for improved metabolic efficiency.

During refeeding, IBAT glycogen over-accumulated before lipid repletion (Fig. 3B), and returned to baseline levels by the time lipids had fully returned to the tissue (Fig. 3D). This same trend has also been noted in WAT by other investigators [28], [29], [62], suggesting that adipocytic glycogen is serving similar purposes in both tissues. The correlation between the return of glycogen to fed-state levels and the total repletion of lipids raises the possibility of a potential interplay between the two substrates. Glycogen may play a role in modulating lipid retention at the substrate level by serving as an intracellular source of glycerol-3-phsophate, improving the re-esterification of recently hydrolyzed FFA, reducing the rate of lipids secreted or oxidized (Fig. 7). If glycogen was serving as a source of substrate for activation of this pathway, it would have a 2-fold effect, increasing the rate of esterification of *de novo*-synthesized fatty acids, as well as the re-esterification of FFA recently hydrolyzed from intracellular stores. The relationship between the over-accumulation of glycogen and improved lipid retention was demonstrated by prior investigations that manipulated glycogen dynamics through use of a transgenic mouse model, which resulted in the marked, permanent over-accumulation of glycogen in EPI and IBAT. Persistent elevation of WAT glycogen levels slowed weight loss following a transition from high-fat diet to normal chow diet through the enhanced retention of free fatty acids in adipocytes [36], [63]. During the present study glycogen over-accumulation in the major WAT and BAT adipose depots may have enabled a similar lipid-retention effect. Such a large store of glycogen, metabolized over the course of 48 hr into TG-precursors (in part), could greatly enhance the survivability of mammals during sporadic nutritional availability, however additional work is needed to validate this supposition.

The pervasiveness of the over-accumulation phenomenon in mice is even more striking considering that these colonies have spent dozens-to-hundreds of generations in laboratory housing without any exposure to acute or chronic caloric stressors. Though the relevance of this glycogen accumulation phenomenon to lipid cycling, heat generation, and/or energy-balance sensing remains speculative, it should be possible to alter rate-limiting glycogen metabolic enzyme activities such as glycogen phosphorylase or glycogen synthase to assess prevailing hypotheses. After these assessments, it might be possible to clearly define any physiological role specific to lipid recovery from starvation, or other critical responses to metabolic stress.
